# The influence of obesity on calf blood flow and vascular reactivity in older adults

**DOI:** 10.1186/1476-5918-6-4

**Published:** 2007-03-26

**Authors:** Luke S Acree, Philip C Comp, Thomas L Whitsett, Polly S Montgomery, Kevin J Nickel, Anette S Fjeldstad, Cecilie Fjeldstad, Andrew W Gardner

**Affiliations:** 1 Department of Health and Exercise Science, University of Oklahoma, Norman, OK, USA; 2 Department of Medicine, Hematology Section, University of Oklahoma Health Sciences Center, Oklahoma City, OK, USA; 3 Department of Medicine, Cardiovascular Section, University of Oklahoma Health Sciences Center, Oklahoma City, OK, USA; 4 Children’s Medical Research Institute (CMRI) Metabolic Research Center, University of Oklahoma Health Sciences Center, Oklahoma City, OK, USA; 5 Department of Veteran Affairs Medical Center, Oklahoma City, OK, USA

## Abstract

**Objective:**

To determine whether differences in vascular reactivity existed among normal weight, overweight, and obese older men and women, and to examine the association between abdominal fat distribution and vascular reactivity.

**Methods:**

Eighty-seven individuals who were 60 years of age or older (age = 69 ± 7 yrs; mean ± SD) were grouped into normal weight (BMI < 25; n = 30), overweight (BMI ≥ 25 and < 30; n = 28), or obese (BMI ≥ 30; n = 29) categories. Calf blood flow (BF) was assessed by venous occlusion strain-gauge plethysmography at rest and post-occlusive reactive hyperemia.

**Results:**

Post-occlusive reactive hyperemia BF was lower (p = 0.038) in the obese group (5.55 ± 4.67 %/min) than in the normal weight group (8.34 ± 3.89 %/min). Additionally, change in BF from rest to post-occlusion in the obese group (1.93 ± 2.58 %/min) was lower (p = 0.001) than in the normal weight group (5.21 ± 3.59 %/min), as well as the percentage change (75 ± 98 % vs. 202 ± 190 %, p = 0.006, respectively). After adjusting for age, prevalence in hypertension and calf skinfold thickness, change in BF values remained lower (p < 0.05) in obese subjects compared to the normal weight subjects. Lastly, the absolute and percentage change in BF were significantly related to BMI (r = -0.44, p < 0.001, and r = -0.37, p < 0.001, respectively) and to waist circumference (r = -0.36, p = 0.001, and r = -0.32, p = 0.002).

**Conclusion:**

Obesity and abdominal adiposity impair vascular reactivity in older men and women, and these deleterious effects on vascular reactivity are independent of conventional risk factors.

## Background

Obesity affects 130 million adults in the United States, and is a major medical concern due to the associated economic and health consequences. Obesity increases the health care costs of older men and women by 33% and 36%, respectively [1,2]. Furthermore, obesity increases the risk of morbidity and mortality, as more than 45% of the total cases of cardiovascular disease and 13% of all deaths occurring in the United States are attributed to obesity [3-7]. Obesity and abdominal fat distribution are associated with endothelial dysfunction [8], which is an initial step in the pathogenesis of atherosclerosis and strongly linked to cardiovascular mortality in the elderly population [9].

Because the vasodilation occurring after a bout of severe hypoxia is dependent on endothelial cell function [10], endothelial function can be assessed by measuring the change in blood flow after vascular occlusion (i.e., vascular reactivity) [11]. In addition to the effect that obesity exerts on the endothelium, it is also associated with hypertension [12], diabetes [13,14], and cardiovascular disease [14-16]. Because these co-morbid conditions impair endothelial function and vascular reactivity [6], it is unclear whether obesity has a direct influence on endothelial function, or whether the co-morbid conditions are the primary mediators.

The purposes of this study were to determine whether differences in vascular reactivity existed among normal weight, overweight, and obese older men and women free of cardiovascular disease, and to examine the association between abdominal fat distribution and vascular reactivity.

## Methods

### Subjects

#### Recruitment

A total of 87 individuals between 60 and 89 years of age participated in this investigation. The subjects were recruited from local newspaper advertisements. Prior to investigation, each subject completed a written informed consent. The study was conducted with the approval of the Institutional Review Board at the University of Oklahoma and the University of Oklahoma, Health Sciences Center.

#### Inclusion and exclusion criteria

To meet the inclusion criteria, subjects were 60 years of age or older, ambulatory, and living independently at home. The exclusion criteria were evaluated by administering a medical history questionnaire and determining ankle/brachial index [17]. Subjects were excluded with the following medical history (or diagnoses): heart or vascular disease, myocardial infarction, cerebrovascular occurrence, peripheral arterial disease (defined by an ankle/brachial index < 0.90 [18]), diabetes, and smoking within the previous year.

### Measurements

#### Demographic information and determination of groups

During a medical history interview, cardiovascular risk factors and co-morbid conditions were assessed. Height and weight were measured using a stadiometer (SECA Corporation; Columbia, MD) and balance scale (SECA Corporation; Columbia, MD), respectively. Body mass index (BMI) was calculated from the height and weight measurements: BMI = weight (kg)/height (m)^2^. Based on the BMI values, subjects were either categorized as normal weight (BMI < 25), overweight (BMI ≥ 25 and < 30), or obese (BMI ≥ 30) [19]. A nine-site sum of skinfold assessment (the total sum for bicep, tricep, chest, subscapular, midaxillary, iliac, abdominal, thigh and calf skinfold sites), a waist circumference, and a waist-to-hip ratio were also collected for all subjects and used for analysis.

#### Peripheral blood flow assessment

Calf blood flow was assessed by venous occlusion strain-gauge plethysmography at rest and following a reactive hyperemic test as previously described [17]. A mercury-in-rubber strain gauge was placed around the calf at the maximal circumference, and blood pressure cuffs were placed around the thigh and ankle. Following 10 minutes of supine rest, the measurement of blood flow was isolated in the calf by inflating the thigh cuff to a venous occlusion pressure of 50 mmHg, and by temporarily occluding arterial blood flow to the foot by inflating the ankle cuff to 200 mmHg. The ankle and thigh cuffs were deflated immediately after the calf blood flow measurement was obtained. The reactive hyperemic test was then performed while patients were in the supine position by inflating a thigh blood pressure cuff to inducing arterial occlusion for three minutes. The thigh blood pressure cuff was inflated to the highest value of either 200 mmHg or 50 mmHg above systolic blood pressure. Post-occlusive reactive hyperemic calf blood flow measurements were obtained within 15 seconds following arterial occlusion. We previously demonstrated that the test-retest intraclass reliability coefficient was R = 0.86 for calf blood flow [20].

### Statistical analysis

Descriptive statistics were computed for the physical characteristics and measured variables. Analysis of variance (ANOVA) was used to assess differences among the normal weight, overweight, and obese groups for continuous variables, and chi-square tests were used to assess differences among the three groups for categorical variables. A post hoc analysis with a Bonferroni correction was done to identify groups that were significantly different from each other. Analysis of covariance (ANCOVA) was used to assess group differences in calf blood flow adjusting for differences in physical and clinical characteristics. Pearson correlation coefficients were also calculated to assess the relationships among vascular reactivity, BMI, waist circumference, and waist-to-hip ratio. All values are reported as the mean ± standard deviation (SD). The level of statistical significance for the study was set at p ≤ 0.05. All statistical analyses were performed with the Statistical Package for the Social Sciences (SPSS, v. 11.5, Chicago, IL) software.

## Results

The physical and clinical characteristics of the subjects in each weight group are shown in Table 1. Group differences (p ≤ 0.05) were found for age, weight, BMI, waist circumference, waist-to-hip ratio, calf skinfold, sum of skinfolds and hypertension. Weight, BMI, and waist circumference were different (p < 0.017) for each pair-wise group comparison, with the normal weight group having the lowest values, the overweight group having intermediate values, and the obese group having the highest values. Additionally, the waist-to-hip ratio was lowest in the normal weight group (p < 0.017), and the sum of skinfolds was greatest in the obese group (p < 0.017).

**Table 1:** Physical and clinical characteristics of normal weight, overweight, and obese subjects. The p value represents the significance among the three weight groups.

	***Normal (n = 30)***	***Overweight (n = 28)***	***Obese (n = 29)***	***p value***
***Age (yrs)***	70 ± 7	72 ± 6	66 ± 5^†^	0.009
***Height (cm)***	168.0 ± 8.7	167.9 ± 8.0	168.4 ± 8.7	0.968
***Weight (Kg)***	64.0 ± 9.0	77.6 ± 8.1*	95.9 ± 14.1*^†^	< 0.001
***Body Mass Index (Kg/m^2^)***	22.6 ± 1.8	27.5 ± 1.5*	33.7 ± 3.7*^†^	< 0.001
***Waist Circumference (cm)***	77.6 ± 8.6	93.0 ± 12.7*	103.5 ± 13.1*^†^	< 0.001
***Waist-to-Hip Ratio***	0.81 ± 0.09	0.91 ± 0.12*	0.89 ± 0.10*	0.001
***Calf Skinfold (mm)***	16 ± 7	18 ± 8	22 ± 12	0.050
***Sum of Skinfolds (mm)***	148 ± 49	177 ± 49	223 ± 70*^†^	< 0.001
***Sex (% Male)***	30	57	48	0.107
***Race (% Caucasian)***	97	89	90	0.602
***Hypertension (%)***	23	32	59	0.008
***Hyperlipidemia (%)***	23	32	31	0.734

Values are means ± standard deviations. * Significant difference from the normal weight group (p < 0.017). ^† ^Significant difference from the overweight group (p < 0.017).

The calf blood flow measurements of the subjects in each weight group are shown in Table 2. Group differences (p < 0.05) were found for post-occlusive reactive hyperemic calf blood flow during the first 15 seconds following the occlusion test, as well as for the absolute and percentage changes in calf blood flow from rest to post-occlusive reactive hyperemia. All three measures were higher in the normal weight group than in the obese group (p < 0.017).

**Table 2:** Calf blood flow of normal weight, overweight, and obese subjects. The p value represents the significance among the three weight groups.

***Measurement***	***Normal (n = 30)***	***Overweight (n = 28)***	***Obese (n = 29)***	***Signif. (p value)***
***Resting Calf Blood Flow*** ***(mL/100 mL/min)***	3.13 ± 1.46	2.60 ± 0.89	3.63 ± 4.41	0.367
***PORH*** ***(mL/100 mL/min)***	8.34 ± 3.89	6.67 ± 3.77	5.55 ± 4.67*	0.038
***Absolute *Δ *in Blood Flow*** ***(mL/100 mL/min)***	5.21 ± 3.59	4.07 ± 3.51	1.93 ± 2.58*	0.001
***% *Δ *in Blood Flow***	202 ± 190	161 ± 148	75 ± 98*	0.006

PORH = post-occlusive reactive hyperemia. Values are means ± standard deviations.

* Significant difference from the normal weight group (p < 0.017).

The calf blood flow measurements of the three groups adjusted for age, hypertension and calf skinfold thickness are shown in Table 3. Group differences (p < 0.05) remained for adjusted values of absolute and percentage changes in calf blood flow from rest to post-occlusive reactive hyperemia. The adjusted absolute change in calf blood flow was lower (p < 0.017) in the obese group than in the normal weight group, and the adjusted percentage change in the obese group was lower (p < 0.017) than in both the normal weight and overweight groups.

**Table 3:** Adjusted values of calf blood flow of normal weight, overweight, and obese subjects. The p value represents the significance among the three weight groups.

***Measurement***	***Normal (n = 30)***	***Overweight (n = 28)***	***Obese (n = 29)***	***Signif. (p value)***
***Resting Calf Blood Flow*** ***(mL/100 mL/min)***	3.09 ± 2.88	2.38 ± 2.82	4.02 ± 3.13	0.159
***PORH*** ***(mL/100 mL/min)***	8.26 ± 4.41	6.61 ± 4.31	5.99 ± 4.79	0.139
***Absolute *Δ *in Blood Flow*** ***(mL/100 mL/min)***	5.18± 3.49	4.23 ± 3.41	1.97 ± 3.80*	0.009
***% *Δ *in Blood Flow***	204 ± 158	176 ± 154	64 ± 172*^†^	0.009

PORH = post-occlusive reactive hyperemia. Values, displayed as means ± standard deviations, were adjusted for age, presence of hypertension, and calf skinfold thickness. * Significant difference from the normal weight group (p < 0.017). ^† ^Significant difference from the overweight group (p < 0.017).

**Figure 1:**
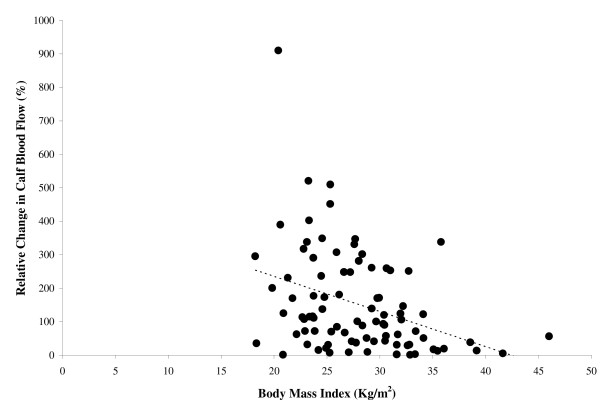
The relationship between body mass index and the percentage change in calf blood flow from rest to post-occlusive reactive hyperemia (r = -0.37, p < 0.001).

**Figure 2:**
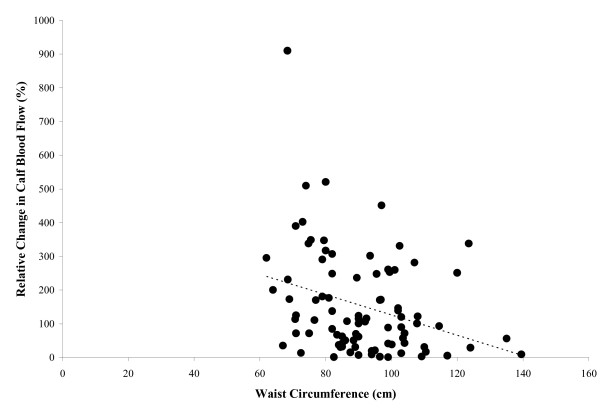
The relationship between waist circumference and percentage change in calf blood flow from rest to post-occlusive reactive hyperemia (r = -0.32, p = 0.002).

None of the anthropometric variables were significantly related (p > 0.05) to calf blood flow obtained at rest. BMI was inversely related to the post-occlusive reactive hyperemic calf blood flow (r = -0.25, p = 0.022), and to the absolute change (r = -0.44, p < 0.001) and percentage change (r = -0.37, p < 0.001) (Figure 1) in calf blood flow from rest to post-occlusive reactive hyperemia. Similarly, waist circumference was inversely related to post-occlusive reactive hyperemic calf blood flow (r = -0.21, p = 0.047), and to the absolute change (r = -0.36, p = 0.001) and percentage change (r = -0.32, p = 0.002) (Figure 2) in calf blood flow from rest to post-occlusive reactive hyperemia.

One subject had a percentage change in calf blood flow that exceeded 900%. We used two different approaches to assess whether this data point had an influence on the relationships shown in Figures 1 and 2. In the first approach, we used non-parametric procedures by calculating the Spearman Rank correlation coefficients. The percentage change in calf blood flow remained significantly related to BMI (r = -0.38, p < 0.001) and to waist circumference (r = -0.33, p = 0.002), which were similar to the coefficients obtained using parametric procedures. The second approach was to repeat the Pearson correlation coefficients after removal of this outlying data point. BMI remained inversely related to the post-occlusive reactive hyperemic calf blood flow (r = -0.23, p = 0.036), as well as the absolute change (r = -0.36, p = 0.001) and percentage change (r = -0.28, p = 0.014) in calf blood flow from rest to post-occlusive reactive hyperemia Additionally, waist circumference remained inversely related to the absolute change (r = -0.33, p = 0.002) and percentage change (r = -0.26, p = 0.019) in calf blood flow from rest to post-occlusive reactive hyperemia. The results from both approaches suggest that the outlying data point had minimal effect on the observed significant associations among these measures.

## Discussion

This investigation compared the reactive hyperemic response to three minutes of arterial occlusion in older adults having a wide range in BMI, and determined if calf blood flow differences among normal weight, overweight, and obese older adults persisted after adjusting for confounders, such as age and hypertension. The primary findings were: (1) the obese group had a blunted change in post-occlusive reactive hyperemic blood flow, indicative of impaired vascular reactivity, than the normal weight group, and (2) the difference in vascular reactivity between the obese and normal weight groups remained significant after controlling for age, hypertension and calf skinfold thickness.

The observation that obesity impairs vascular reactivity supports previous studies in young and healthy adults [8,16,21], and extends this finding to obese older adults who are free of overt cardiovascular disease. This suggests that obesity-mediated vascular dysfunction in older adults is due to impairment in endothelial function [8,16]. Our results are further supported by a report that found an inverse correlation between obesity and endothelial-independent vasodilation [22] in a small sample of older adults with diabetes. Collectively, these findings suggest that obesity has a detrimental impact on endothelial function in older adults with and without diabetes.

Besides the negative consequences of obesity on vascular function in older populations, obesity-mediated alterations in endothelial function are evident even in young adults. A lower response in endothelial-dependent vasodilation and forearm blood flow after an infusion of acetylcholine (ACh) is observed in obese, young adults compared to overweight and normal weight young adults [8]. Accumulation of abdominal fat is the primary factor for endothelial dysfunction [21]. Interestingly, endothelial function may be positively altered following a weight loss program. A prospective investigation found ACh-stimulated forearm blood flow improved following a reduction in body size and waist circumference [16]. In speculation, a program designed to decrease adiposity may improve endothelial function in older, obese adults.

The increased prevalence of chronic vascular complications consistently shown in the aging population [23,24] further complicates the association between obesity and endothelial function. The development of endothelial dysfunction and, ultimately, atherosclerosis has specifically been linked to diabetes [25], hypertension [26], and hypercholesterolemia [27], all of which increase in prevalence with advancing age [28]. Additionally, lifestyle behaviors such as physical inactivity and smoking, impair endothelial function and initiate atherosclerotic processes [23,24,29-31]. The lack of control of these co-morbid conditions leaves previous results inconclusive regarding the independent role of obesity in the pathogenesis of endothelial dysfunction. The current investigation attempted to minimize these confounding factors by excluding subjects with cardiovascular disease, diabetes, and a history of smoking during the previous year. Furthermore, vascular reactivity measurements were adjusted for group differences in age, prevalence of hypertension and calf skinfold thickness. These approaches improve the ability to determine the influence of obesity on vascular reactivity.

Finally, subcutaneous body fat, as assessed by the sum of skinfolds, did not show any relationship to blood flow or to vascular reactivity in this population. In contrast, the central distribution of adipose tissue, as assessed by waist circumference, was inversely related to vascular reactivity of these older adults. Taken collectively, these results suggest that visceral adiposity may have a more detrimental influence on vascular reactivity (i.e., endothelial dysfunction) than subcutaneous fat. Our findings are supported by a previous observation that impairment in flow-mediated, endothelium-dependent vasodilation of the brachial artery occurs with visceral obesity, rather than with subcutaneous obesity [32].

One limitation to this study is the cross-sectional design which does not establish a causal link between obesity and impaired vascular reactivity. Intervention and longitudinal studies that track changes in fat mass and vascular reactivity are necessary to better determine their association. Although we normalized the blood flow measures according to subcutaneous fat of the calf, as estimated by the calf skinfold, we did not measure intra-muscular fat. Therefore, it is possible that the difference in blood flow among the groups is partially attributed to differences in intra-muscular fat of the calf. Additionally, the measurement of vascular reactivity by the method of reactive hyperemia assesses the vasodilatory function of both the endothelium and vascular smooth muscle, and therefore is only an indirect assessment of endothelial function. The lack of physical activity measurement is another limitation to this study. Physical activity status is associated with blood flow [24] and endothelial-dependent vasodilatory function [29] and, thus, should be considered in future studies examining the influence of obesity and abdominal fat on vascular reactivity.

Although our investigation minimized confounding factors by excluding for cardiovascular disease and controlling for many primary risk factors of atherosclerosis, we did not adjust for the possible influence of secondary risk factors, such as C-reactive protein or other inflammatory markers [33,34]. Furthermore, a medical history was used to exclude participants with diagnosed cardiovascular disease and diabetes, but those with undiagnosed conditions may not have been identified. That said, the possibility exists that our self-report method of revealing existing disease does allow for an underestimated prevalence of diabetes. Lastly, our assessment of adiposity was limited to BMI and anthropometric measures rather than more precise measurements of body fat and displacement of adiposity.

In conclusion, obesity and abdominal adiposity impair vascular reactivity in older men and women, and these deleterious effects on vascular reactivity are independent of conventional risk factors. Consequently, impaired vascular reactivity may increase the risk for subsequent cardiovascular complications in older, obese adults.

## Competing interests

The author(s) declare that they have no competing interests.

## Authors' contributions

LSA conceived and designed the study, acquired data, performed the statistical analyses and their interpretation, and drafted each manuscript version. PCC, TLW and PSM assisted with subject recruitment and manuscript revision. KJN, ASF, CF assisted with data collection and manuscript revision. AWG conceived and designed the study, assisted with statistical analyses and their interpretation, and drafted the manuscript. All authors read and approved the final manuscript.
